# Social norms, attitudes and access to modern contraception for adolescent girls in six districts in Uganda

**DOI:** 10.1186/s12913-021-07060-5

**Published:** 2021-10-02

**Authors:** Paul Bukuluki, Peter Kisaakye, Maxime Houinato, Adekemi Ndieli, Evelyn Letiyo, Dan Bazira

**Affiliations:** 1 School of Social Sciences, Makerere University, Kampala, Uganda; 2 School of Statistics and Planning, Makerere University, Kampala, Uganda; 3 UN Women, Kampala, Uganda

**Keywords:** Social norms, Attitudes, Access and Use of Contraception, Adolescents, Uganda

## Abstract

**Background:**

Social norms continue to be entrenched in Uganda. Understanding social norms helps to uncover the underlying drivers that influence attitudes and behavior towards contraceptive access and use. This study therefore seeks to investigate the factors that influence the social norm – access to contraception by adolescent girls – in six districts in Uganda.

**Data and methods:**

Using data from a community cross-sectional survey in six districts (Amudat, Kaberamaido, Kasese, Moroto, Tororo and Pader) in Uganda, a binary logistic regression model was fitted to examine the variation in individual beliefs and socio-economic and demographic factors on ‘allowing adolescent girls to access contraception in a community’ – we refer to as a social norm.

**Results:**

Results demonstrate that a higher proportion of respondents hold social norms that inhibit adolescent girls from accessing contraception in the community. After controlling for all variables, the likelihood for adolescent girls to be allowed access to contraception in the community was higher among respondents living in Kaberamaido (OR = 2.58; 95 %CI = 1.23–5.39), Kasese (OR = 2.62; 95 %CI = 1.25–5.47), Pader (OR = 4.35; 95 %CI = 2.15–8.79) and Tororo (OR = 9.44; 95 %CI = 4.59–19.37), those aged 30–34 years likely (OR = 1.73; 95 %CI = 1.03–2.91). However, the likelihood for respondents living in Moroto to agree that adolescent girls are allowed to access contraception was lower (OR = 0.27; 95 %CI = 0.11–0.68) compared to respondents living in Amudat. Respondents who were not formally employed (OR = 0.63; 95 %CI = 0.43–0.91), and those who agreed that withdrawal prevents pregnancy (OR = 0.45; 95 %CI = 0.35–0.57) were less likely to agree that adolescent girls are allowed to access contraception in the community. Respondents who agreed that a girl who is sexually active can use contraception to prevent unwanted pregnancy (OR = 1.84; 95 %CI = 1.33–2.53), unmarried women or girls should have access to contraception (OR = 2.15; 95 %CI = 1.61–2.88), married women or girls should have access to contraception (OR = 1.55; 95 %CI = 0.99–2.39) and women know where to obtain contraception for prevention against pregnancy (OR = 2.35; 95 %CI = 1.19–4.65) were more likely to agree that adolescent girls are allowed to access contraception.

**Conclusions:**

The findings underscore the need for context specific ASRH programs that take into account the differences in attitudes and social norms that affect access and use of contraception by adolescents.

## Introduction

Several scholars have made an attempt to define and explain the concept of social norms. Most of the definitions lean towards their academic disciplines. Some scholars especially social scientists have argued that social norms are context-dependent rules of what is perceived as obligatory, appropriate, and acceptable behaviour shared by people in the same group, community or society [1–3].

In this paper we lean more towards the sociological explanation of social norms that conceptualizes social norms as customary or unwritten rules that govern people’s behavior in a community, that are enforced by relevant reference groups [5–7]. Social norms can be classified under three main perspectives: (1) Social norms as behavioral regularities – which result from repeating behaviors [8, 9]. (2) Pluralistic ignorance – where individuals think that their personal beliefs, ideas or feelings are different from others but their public behavior should be the same [10–12] and (3) Social norms as social beliefs – governed by the behavior of other people in a community [13, 14].

Social norms have also been categorized into descriptive norms and injunctive norms [15]. Descriptive norms are conceptualized to refer to one’s belief about what others typically do in a given situation while injunctive norms are one’s belief about what actions other people approve and disapprove [15]. More recent publications by Ben Cislaghi and Lori Heise have cautioned practitioners to take into consideration what they call the “eight learnings” that practitioners should reflect upon as they plan to integrate a social norms perspective in their interventions [16]. These learnings include: Social norms and attitudes are different; Social norms and attitudes can coincide; Protective norms can offer important resources for achieving effective social improvement in people’s health-related practices; Harmful practices are sustained by a matrix of factors that need to be understood in their interactions;

The prevalence of a norm is not necessarily a sign of its strength; Social norms can exert both direct and indirect influence; Publicizing the prevalence of a harmful practice can make things worse; and People-led social norm change is both the right and the smart thing to do [16]. These learnings underscore the complexity of the interaction between social norms and other structural and institutional factors that regulate behaviour especially sexual and reproductive health practices in different contexts. They also point to the need to appreciate the notion and reality that some social norms can be harmful while others are not [13, 16] underscoring the need to identify positive cultural resources that can serve as building blocks for promoting sexual and reproductive health [16]. Social norms can aid in understanding the reproductive health of adolescents or young girls in developing countries [6], but social norms also influence someone’s attitudes and behavior [17]. For example, social norms can influence whether and how people can access contraception. This paper seeks to examine the variation in terms of individual beliefs and socio-economic and demographic factors among respondents in six districts in Uganda on ‘allowing adolescent girls to access contraception in a community’ – we refer to as a social norm. In this paper, we refer to adolescent girls as girls in the age group 10–19 years [18]. This paper uses the terms ‘contraception’ and ‘family planning’ interchangeably to mean the same thing. Using the definition from the ‘dictionary of demography’, family planning refers to any attempt to control the number and spacing of children [19].

Adolescents constitute the largest proportion of the world’s population [20]. Girls below the age of 18 years are a critical age group that needs to be supported along their life trajectories [20–23]. However, adolescent girls tend to be disproportionately affected by the gendered social norms more than boys [24–28]. Social norms have been observed to impede access and use of modern contraception particularly among adolescent girls [22]. As a result adolescent girls face a problem meeting their sexual and reproductive health needs [21]. For example, some social norms prevent adolescent girls from making decisive and independent decisions regarding their reproductive health such as access or the use of contraception [29]. The problem is compounded with early initiation of sexual intercourse [30]. Such practices would make adolescent girls get unwanted pregnancies [31], sexually transmitted diseases [32] or early marriages [33] that would have been averted, if they had the freedom to make independent choices about the use of contraception [30, 34, 35]. For example, in Ethiopia, social norms force adolescent girls to enter into early marriages to preserve their virginity, but also prevent out-of-marriage births [21]. In Uganda, about half (49 %) of girls get married by the age of 18 years [36].

Recent studies [4, 24] have emphasized that social norms can have an effect on adolescents and that ultimately shapes how adolescents manage their reproductive health spheres later in life. That is, social norms can have an effect on the way adolescent girls make sexual and reproductive health choices. This is because social norms are adopted and internalized when people are still young [21].

While there is a large body of evidence on social norms or access and use of contraception, little is known about the effect of social norms on reproductive health, such as access and use of contraception in Uganda [4, 22, 24]. Yet, understanding the sociocultural context can help in designing strategic and positive interventions aimed at improving sexual and reproductive health behavior of adolescent girls [21, 25]. For example, some sociocultural contexts do not permit free discussions related to sexual and reproductive health with young people [30, 37–39]. Moreover, most sexual and reproductive health interventions tend to focus on older women while neglecting adolescent girls that are dealing with emerging issues about sexual and reproductive health such as sexuality, fertility or puberty [40].

This study therefore seeks to investigate the factors that influence the social norm – access to contraception by adolescent girls – in six districts in Uganda. We answer this by examining the socio-economic and demographic as well as individual beliefs/attitudes that influence allowing adolescent girls to have access to contraception. We expect that individuals with positive attitudes or beliefs about sexual and reproductive health behavior are more likely to agree that adolescent girls are allowed to access contraception in their community. The results in this study can inform policies that can be scaled-up or reformulated to challenge the status-quo which can help avert early marriages, unwanted pregnancies among young people. Such policies can incorporate improving young girls’ agency and ability to make informed sexual and reproductive health goals [21]. Ultimately, the results in this study can help in the design of norm-focused interventions rather than individual-focused interventions in promoting better sexual and reproductive health behavior among adolescents [16, 23, 41].

## Context

The six districts in the study are; Amudat, Kaberamaido, Kasese, Moroto, Tororo and Pader. Three of the districts (Amudat, Moroto, and Pader) are located in the northern region, two districts (Kaberamaido and Tororo) in the eastern region and Kasese district in the western region. Amudat and Moroto are located in Karamoja sub-region – which is one of the least developed regions in Uganda [42, 43]. Agriculture forms the main economic activity in the study districts [44]. Social norms and patriarchy systems continue to be entrenched in the study districts – and often prohibit girls or women from making independent life choices [45, 46].

According to the 2014 National Population and Housing Census, Amudat district has a total population of about 105,769 people. The total population of adolescents aged 0–17 years is 61,299 people. The total number of ever married females aged 10–19 years is 1,614 [47]. Moroto district has a total population of about 103,432 people. The total population of adolescents aged 0–17 years is 54,975 people. The total number of ever married females aged 10–19 years is 1,933 [48]. Pader district has a total population of about 178,004 people. The total population of adolescents aged 0–17 years is 102,812 people. The total number of ever married females aged 10–19 years is 3,569 [49]. Kaberamaido district has a total population of about 215,026 people. The total population of adolescents aged 0–17 years is 126,093 people. The total number of ever married females aged 10–19 years is 3,694 [50]. Tororo district has a total population of about 517,080 people. The total population of adolescents aged 0–17 years is 291,043 people. The total number of ever married females aged 10–19 years is 10,155 [51], and Kasese district has a total population of about 694,987 people. The total population of adolescents aged 0–17 years is 385,664 people. The total number of married females aged 10–19 years is 12,665 [52].

## Data and methods

### Source of data

 All methods were carried out in accordance with relevant guidelines and regulations. The data used in this study come from a community cross-sectional survey. The survey aimed at collecting data that can be used to assess social norms and sexual and reproductive health rights (SRHRs), violence against women and girls (VAWG) and harmful practices (HP).

### Study sites and population

Data collection took place in six districts of Uganda (Amudat, Kaberamaido, Kasese, Moroto, Tororo and Pader) where the EU Spotlight Initiative to eliminate violence against women and girls is implemented [53, 54].

**Fig. 1:**
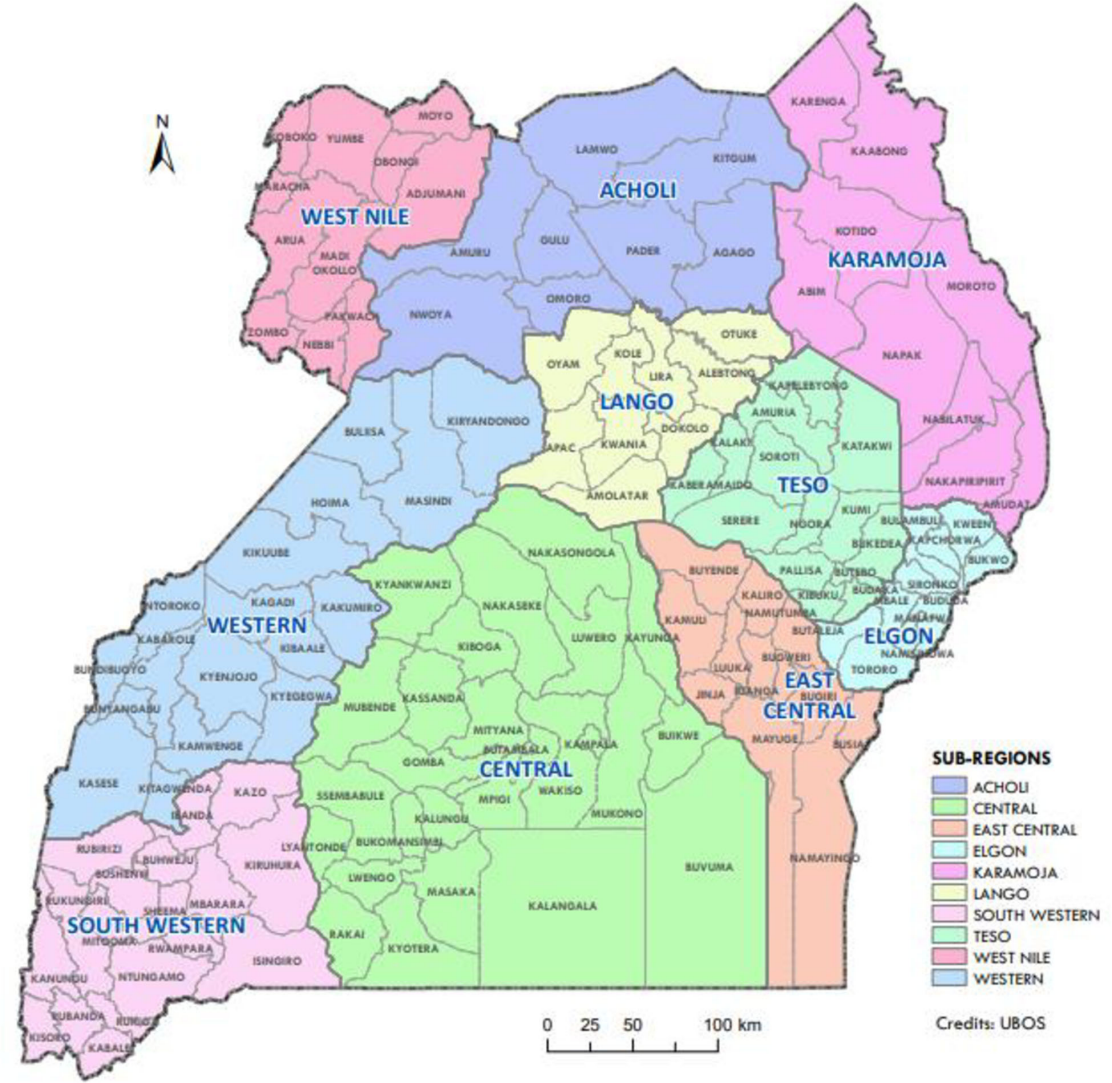
A map of Uganda showing the location of the districts in the study

Figure 1 shows the location of the districts in the study. Amudat district is located in the Karamoja sub-region in the northern region, Kaberamaido in the Teso sub-region in the eastern region, Kasese in Rwenzururu sub-region in the western region, Moroto in Karamoja sub-region in the northern region, Tororo in the Elgon sub-region in the eastern region, and Pader in Acholi sub-region in the northern region [55].

### Sampling

A list of locations within each district was developed together with the community leaders. The community survey was based on a stratified two-stage cluster design, with districts as study domains. In the first stage, a simple random sample of ten villages was taken using probability proportional to size (approximate number of households) sampling in each district. In the second stage, a list of locations within the selected villages was classified into small and large areas or routes or path junctions of traffic. A simple random sample of 3–5 small locations and 2–3 large locations were chosen. Finally, a systematic random sampling approach was used to recruit respondents by recruiting every third person wearing a certain color of clothes. Using the formula of simple random sampling with proportions, p = 0.05495, q = 0.94505, z = 2.58, margin of error=+/-1 % at 99 % level of significance, we estimated a sample of 3456 respondents. The response rate was 99 % since the actual sample size was 3427 individuals.

### Data collection

Data collection took place in August-September 2019 by a team of four well trained research assistants per district (two females and two males). Interviews were done in a private setting, and responses were electronically recorded using a mobile tablet programmed with a quantitative survey tool. Given the sensitivity of some of the questions included in the survey, female interviewers were required to interview only female respondents while male interviewers were required to interview only male respondents. A pre-test was done prior to the actual data collection exercise. This was aimed to test the validity and reliability of the questionnaire and lessons learned during the pre-test were incorporated in the final revision of the quantitative questionnaire.

### Variables

The socio-economic and demographic variables that were included in the study are sex (female or male), age in single years, current marital status (single, currently married, widowed or separated or divorced), level of education (no education, primary, secondary, vocational or university), religion (Catholic, Protestant, Born again, Moslem, SDA, Traditional) and formal employment in the last three months preceding the survey (no or yes).

The study also collected information on people’s attitudes/ beliefs about access and use of contraception: Consistent use of the condom prevents pregnancy, It is not easy for women below 20 years to get pregnant, Belief that withdrawal prevents pregnancy, Women or girls have a right to access family planning methods, A girl who is sexually active can use contraception to prevent unwanted pregnancy, Unmarried women or girls should have access to contraception, Married women or girls should have access to contraception, Women know where to obtain contraception for prevention against pregnancy, Women who carry condoms are promiscuous, and It is not good for girls to know about sexual matters because they will get spoilt. Responses to each of these attitudinal questions were disagree or agree.

Respondents were asked whether adolescent girls are allowed to access contraception in the community. Responses to this question were either a yes or no. This question was used to measure the social norm that influences access and use of contraception in communities – as the outcome variable.

### Data analysis

The Stata statistical software was used to analyze the data [56]. Results were presented at the univariate, bivariate and multivariable level of analyses. Frequencies were estimated at the univariate level of analysis to show the distribution of respondents. A Chi-square test was estimated at the bivariate level to measure the relationship between selected variables and access to contraception by adolescent girls in the community. At the multivariable level of analysis, a binary logistic regression model was fitted to examine the factors that are associated with access to contraception by adolescent girls in the community.

### Ethical considerations

Permission to conduct the study was granted by the School of Social Sciences at Makerere University and Uganda National Council for Science and Technology (SS4951). All respondents who participated in the survey provided verbal informed consent before the interview could start. Respondents were assured of utmost safety, confidentiality, voluntary participation and withdraw from the survey without any penalty.

## Results

### Characteristics of the respondents

Table 1 shows the distribution of respondents in the survey by socio-economic and demographic factors. About two-thirds (66 %) of respondents were females. Four out of every five respondents (80 %) were currently married at the time of the survey. About 4 out of every 10 respondents (43 %) had primary education. Half (50 %) of the respondents belonged to the Catholic religious faith. Table 1 shows that about five out of every six respondents (85 %) were not formally employed in the last three months preceding the survey.

**Table 1:** Distribution of respondents by socio-economic and demographic factors

Social economic and demographic variables	Number	Percent
Sex		
Male	1159	33.8
Female	2268	66.2
Age of respondent		
15–19	349	10.2
20–24	576	16.8
25–29	674	19.7
30–34	558	16.3
35–39	462	13.5
40–44	349	10.2
45+	459	13.4
Current marital status		
Single	466	13.6
Married	2740	79.9
Widowed/ Divorced/ Separated	221	6.5
Education		
No education	1005	29.3
Primary	1491	43.5
Secondary	707	20.6
Vocational/ University	224	6.5
Religion		
Catholic	1735	50.6
Born again	556	16.2
Protestant	994	29
Traditional	51	1.5
Moslem	54	1.6
SDA	37	1.1
Formally employed in last 3 months		
No	2904	84.7
Yes	523	15.3
Study district		
Amudat	566	16.5
Kaberamaido	571	16.7
Kasese	583	17.0
Moroto	557	16.2
Pader	576	16.8
Tororo	574	16.8
Total	3427	100

Note: SDA = Seventh Day Adventist. Missing cases are not shown. Figures may not add up to 100 % due to rounding errors

Table 2 shows the distribution of respondents by attitudes about access and use of contraception. The majority agreed that consistent use of the condom prevents pregnancy (80 %), women and girls have a right to access family planning methods (60 %), a girl who is sexually active can use contraception to prevent unwanted pregnancy (60 %), married women or girls should have access to contraception (73 %), women know where to obtain contraception for prevention against pregnancy (84 %), women who carry condoms are promiscuous (75 %) and it is not good for girls to know about sexual matters because they will get spoilt (70 %). Table 2 also shows that about half (50 %) of the respondents agreed that withdrawal prevents pregnancy and unmarried women or girls should have access to contraception.

**Table 2:** Distribution of respondents by beliefs about access and use of contraception

Beliefs about access and use of contraception	Number	Percent
Consistent use of the condom prevents pregnancy		
Disagree	694	20.3
Agree	2733	79.8
It is not easy for women below 20 years to get pregnant		
Disagree	2900	84.6
Agree	527	15.4
Belief that withdrawal prevents pregnancy		
Disagree	1155	50.9
Agree	1113	49.1
Women or girls have a right to access family planning methods		
Disagree	1359	39.7
Agree	2068	60.3
A girl who is sexually active can use contraception to prevent unwanted pregnancy		
Disagree	1357	39.6
Agree	2070	60.4
Unmarried women or girls should have access to contraception		
Disagree	1768	51.6
Agree	1659	48.4
Married women or girls should have access to contraception		
Disagree	926	27.0
Agree	2501	73.0
Women know where to obtain contraception for prevention against pregnancy		
Disagree	540	15.8
Agree	2887	84.2
Women who carry condoms are promiscuous		
Disagree	832	24.5
Agree	2563	75.5
It is not good for girls to know about sexual matters because they will get spoilt		
Disagree	1018	29.8
Agree	2401	70.2
Total	3427	100

Note: Missing cases are not shown. Figures may not add up to 100 % due to rounding errors

### Relationship between selected factors and access to contraception by adolescent girls in community

Table 3 shows that respondents were significantly different by access to contraception by adolescent girls in the community by age of the respondent (*p* < 0.01), current marital status (*p* < 0.01), level of education (*p* < 0.001), and religion (*p* < 0.001). Overall, the results shown in Table 3 indicate that the majority of respondents (73 %) agreed to the social norm that adolescent girls are not allowed to access contraception. However, the proportion of currently married or ever married (widowed, divorced, separated) respondents who stated that adolescent girls are not allowed to access contraception is higher than single respondents. Results indicate that respondents were significantly different by access to contraception by adolescent girls in the community by study district (*p* < 0.001). A higher proportion of respondents who reported that adolescent girls are not allowed access to contraception is observed in all countries but Tororo.

**Table 3:** Relationship between social-economic and demographic factors and access to contraception in the community

Social economic and demographic variables	Adolescent girls are allowed to access contraception in this community		Chi-square (P-value)	Total
	No	Yes		
Sex			1.47 (0.226)	
Male	72.2	27.8		100
Female	74.1	25.9		100
Age of respondent			16.98 (0.009)***	
15–19	70.1	29.9		100
20–24	70.0	30.0		100
25–29	76.9	23.2		100
30–34	73.3	26.7		100
35–39	77.8	22.2		100
40–44	74.7	25.3		100
45+	70.0	30.0		100
Current marital status			14.13 (0.001)***	
Single	66.3	33.7		100
Married	74.5	25.5		100
Widowed/ Divorced/ Separated	75.0	25.0		100
Education			233.82 (0.000)****	
No education	91.3	8.7		100
Primary	65.2	34.8		100
Secondary	66.3	33.7		100
Vocational/ University	71.0	29.0		100
Religion			57.29 (0.000)****	
Catholic	69.5	30.5		100
Born again	80.8	19.2		100
Protestant	76.5	23.5		100
Traditional	93.9	6.1		100
Moslem	51.9	48.2		100
SDA	70.3	29.7		100
Formally employed in last 3 months			0.50 (0.477)	
No	73.2	26.8		100
Yes	74.7	25.3		100
Study district			727.53 (0.000)****	
Amudat	95.0	5.0		100
Kaberamaido	73.4	26.6		100
Kasese	79.1	20.9		100
Moroto	96.0	4.0		100
Pader	61.6	38.4		100
Tororo	36.6	63.4		100
Total	73.5	26.6		100

Note: ***=*p* < 0.01; ****=*p* < 0.001. SDA = Seventh Day Adventist. Missing cases are not shown. Figures may not add up to 100 % due to rounding errors

Table 4 shows that respondents were significantly different by access to contraception by adolescent girls in the community by all attitudes but ‘it is not easy for women below 20 years to get pregnant’. However, the proportion of respondents that agreed that adolescent girls are not allowed to access contraception in the community by different attitudes such as ‘consistent use of the condom prevents pregnancy’ (69 %), ‘women or girls have a right to access family planning methods’ (63 %), ‘a girl who is sexually active can use contraception to prevent unwanted pregnancy’ (63 %), ‘unmarried women or girls should have access to contraception’ (57 %), ‘married women or girls should have access to contraception’ (67 %) and ‘women know where to obtain contraception for prevention against pregnancy’ (70 %) was lower than the proportion that disagreed.

**Table 4:** Relationship between beliefs about access and use of contraception and access to contraception in the community

Beliefs about access and use of contraception	Adolescent girls are allowed to access contraception in this community		Chi-square (P-value)	Total
	No	Yes		
Consistent use of the condom prevents pregnancy			126.61 (0.000)****	
Disagree	90.4	9.6		100
Agree	69.2	30.8		100
It is not easy for women below 20 years to get pregnant			0.02 (0.886)	
Disagree	73.5	26.5		100
Agree	73.2	26.8		100
Belief that withdrawal prevents pregnancy			5.75 (0.016)**	
Disagree	71.9	28.1		100
Agree	76.4	23.7		100
Women or girls have a right to access family planning methods			298.25 (0.000)****	
Disagree	89.6	10.4		100
Agree	62.9	37.1		100
A girl who is sexually active can use contraception to prevent unwanted pregnancy			296.94 (0.000)****	
Disagree	89.6	10.4		100
Agree	62.9	37.1		100
Unmarried women or girls should have access to contraception			427.09 (0.000)****	
Disagree	88.6	11.4		100
Agree	57.3	42.7		100
Married women or girls should have access to contraception			213.44 (0.000)****	
Disagree	91.6	8.4		100
Agree	66.8	33.3		100
Women know where to obtain contraception for prevention against pregnancy			113.76 (0.000)****	
Disagree	92.2	7.9		100
Agree	70.0	30.0		100
Women who carry condoms are promiscuous			31.34 (0.000)****	
Disagree	65.9	34.1		100
Agree	75.8	24.2		100
It is not good for girls to know about sexual matters because they will get spoilt			48.74 (0.000)****	
Disagree	65.3	34.7		100
Agree	76.8	23.2		100
Total	73.5	26.6		

Note: *=*p* < 0.10; **=*p* < 0.05; ***=*p* < 0.01; ****=*p* < 0.001. Missing cases are not shown. Figures may not add up to 100 % due to rounding errors

### Factors associated with access to contraception by adolescent girls in the community

**Fig. 2:**
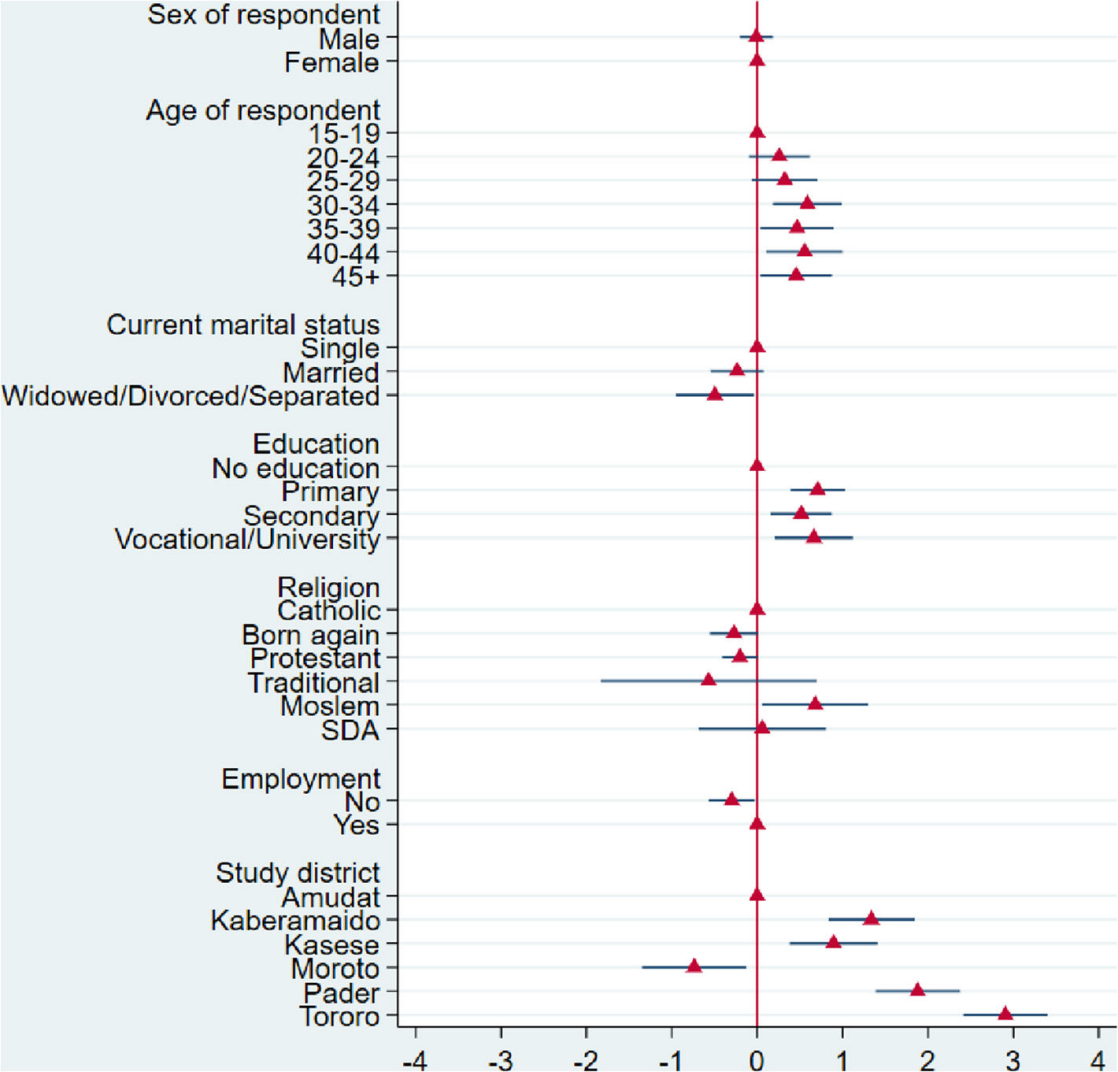
Socio-economic and demographic factors associated with access to contraception by adolescent girls in a community (Model 1)

The results shown in Fig. 2 depict estimates from Model 1 that controlled for only socio-economic and demographic factors that influence the social norm (access to contraception by adolescent girls in the community). Figure 2 shows that respondents aged 30–34 years (OR = 1.80; 95 %CI = 1.20–2.69), 35–39 years (OR = 1.59; 95 %CI = 1.04–2.45), 40–44 years (OR = 1.74; 95 %CI = 1.11–2.71) and 45 or more years (OR = 1.58; 95 %CI = 1.04–2.39) were more likely to agree that adolescent girls are allowed to access contraception in the community than respondents aged 15–19 years. Ever married (widowed, divorced, separated) (OR = 0.61; 95 %CI = 0.38–0.96) respondents were less likely to agree that adolescent girls are allowed to access contraception in the community than single respondents.

Figure 2 shows that respondents with primary (OR = 2.03; 95 %CI = 1.48–2.79), secondary (OR = 1.67; 95 %CI = 1.17–2.39) or vocational/university (OR = 1.94; 95 %CI = 1.23–3.06) were more likely to state that adolescent girls are allowed to access contraception in the community than respondents with no education. Muslims (OR = 1.97; 95 %CI = 1.06–3.66) were more likely to mention that adolescent girls in the community are allowed to access contraception than the Catholics. Respondents with no formal employment in the last three months preceding the survey were less likely (OR = 0.74; 95 %CI = 0.57–0.97) to mention that adolescent girls are allowed to access contraception in the community than their counterparts that were not formally employed. Respondents in Kaberamaido (OR = 3.81; 95 %CI = 2.30–6.31), Kasese (OR = 2.44; 95 %CI = 1.46–4.09), Pader (OR = 6.56; 95 %CI = 4.01–10.73) and Tororo (OR = 18.29; 95 %CI = 11.17–29.95) were more likely to mention that adolescent girls are allowed to access in the community contraception while respondents in Moroto (OR = 0.48; 95 %CI = 0.26–0.88) were less likely to mention that adolescent girls are allowed to access contraception in the community compared to respondents in Amudat.

**Fig. 3:**
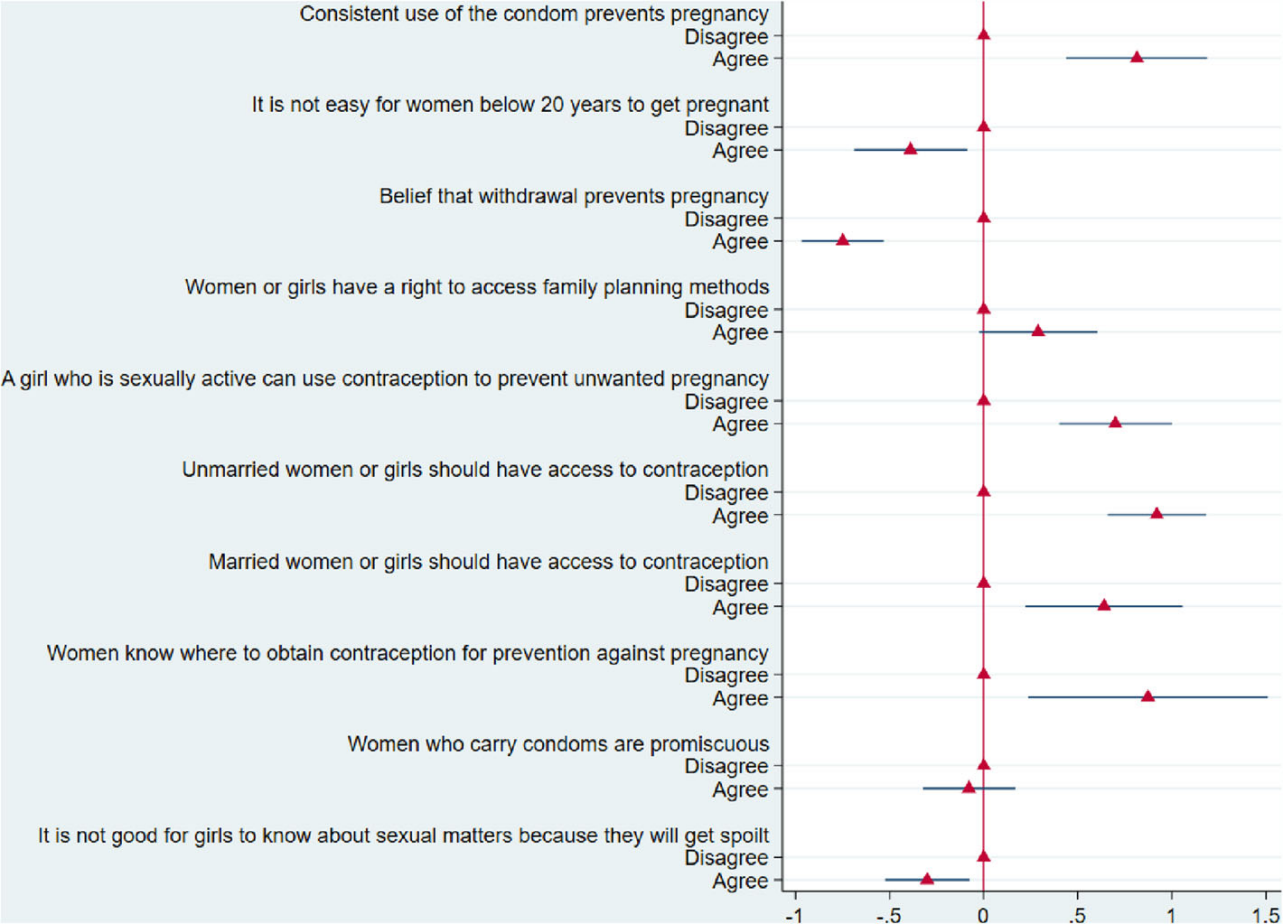
Beliefs about access and use of contraception associated with access to contraception in a community (Model 2)

Figure 3 shows results from Model 2 that controlled for only individual attitudes. Respondents who agreed that consistent use of the condom prevents pregnancy (OR = 2.26; 95 %CI = 1.55–3.28), a girl who is sexually active can use contraception to prevent unwanted pregnancy (OR = 2.01; 95 %CI = 1.49–2.72), unmarried women or girls should have access to contraception (OR = 2.51; 95 %CI = 1.93–3.26), married women or girls should have access to contraception (OR = 1.89; 95 %CI = 1.25–2.88) and women know where to obtain contraception for prevention against pregnancy (OR = 2.39; 95 %CI = 1.27–4.53) were more likely to agree that adolescent girls are allowed to access contraception in the community than respondents who disagreed to any of the beliefs.

The results in Fig. 3 show that respondents who agreed that is it not easy for women below 20 years to get pregnant (OR = 0.68; 95 %CI = 0.50–0.91), withdrawal prevents pregnancy (OR = 0.47; 95 %CI = 0.38–0.59) and it is not good for girls to know about sexual matters because they will get spoilt (OR = 0.74; 95 %CI = 0.59–0.93) were less likely to agree that adolescent girls in the community are allowed to access contraception than their counterparts who disagreed to any of the attitudinal statements.

Table 5 shows results from the full model (Model 3) – that controlled for all variables considered in this study. When all variables are considered, the results indicate that respondents aged 30–34 years were more likely (OR = 1.73; 95 %CI = 1.03–2.91) to agree that adolescent girls are allowed to access contraception in the community than respondents aged 15–19 years. Respondents who were not formally employed in the last three months preceding the survey (OR = 0.63; 95 %CI = 0.43–0.91) were less likely to agree that adolescent girls are allowed to access contraception in the community than respondents who were formally employed. The results in Table 5 show a similar pattern to the one in Fig. 3. That is, respondents who agreed that withdrawal prevents pregnancy (OR = 0.45; 95 %CI = 0.35–0.57) were less likely to agree that adolescent girls are allowed to access contraception in the community than those that disagreed.

Respondents who agreed that a girl who is sexually active can use contraception to prevent unwanted pregnancy (OR = 1.84; 95 %CI = 1.33–2.53), unmarried women or girls should have access to contraception (OR = 2.15; 95 %CI = 1.61–2.88), married women or girls should have access to contraception (OR = 1.55; 95 %CI = 0.99–2.39) and women know where to obtain contraception for prevention against pregnancy (OR = 2.35; 95 %CI = 1.19–4.65) were more likely to agree that adolescent girls are allowed to access contraception in the community than their counterparts who disagreed. The results in Table 5 show that the likelihood for adolescent girls to be allowed access to contraception in the community was higher in Kaberamaido (OR = 2.58; 95 %CI = 1.23–5.39), Kasese (OR = 2.62; 95 %CI = 1.25–5.47), Pader (OR = 4.35; 95 %CI = 2.15–8.79) and Tororo (OR = 9.44; 95 %CI = 4.59–19.37). However, the likelihood for respondents living in Moroto to agree that adolescent girls are allowed to access contraception was lower (OR = 0.27; 95 %CI = 0.11–0.68) compared to respondents living in Amudat.

**Table 5:** Socio-economic, demographic factors, and beliefs about access and use of contraception associated with access to contraception in a community (Model 3)

Variable	Odds Ratio (95 %CI)
Sex (RC = Male)	
Female	-
Age of respondent (RC = 15–19)	
20–24	1.32 (0.82–2.13)
25–29	1.33 (0.81–2.19)
30–34	1.73** (1.03–2.91)
35–39	1.12 (0.64–1.96)
40–44	1.34 (0.75–2.39)
45+	1.32 (0.76–2.30)
Current marital status (RC = Single)	
Married	0.65 (0.42–1.01)
Widowed/ Divorced/ Separated	0.66 (0.37–1.19)
Education (RC = No education)	
Primary	1.38 (0.94–2.01)
Secondary	1.18 (0.76–1.84)
Vocational/ University	1.13 (0.58–2.23)
Religion (RC = Catholic)	
Born again	0.71 (0.49–1.02)
Protestant	0.83 (0.63–1.08)
Traditional	0.37 (0.04–3.01)
Moslem	2.21 (0.97–5.02)
SDA	1.30 (0.50–3.38)
Formally employed in last 3 months (RC = Yes)	
No	0.63** (0.43–0.91)
Consistent use of the condom prevents pregnancy (RC = Disagree)	
Agree	1.17 (0.76–1.81)
It is not easy for women below 20 years to get pregnant (RC = Disagree)	
Agree	0.87 (0.62–1.22)
Belief that withdrawal prevents pregnancy (RC = Disagree)	
Agree	0.45**** (0.35–0.57)
Women or girls have a right to access family planning methods (RC = Disagree)	
Agree	1.18 (0.85–1.65)
A girl who is sexually active can use contraception to prevent unwanted pregnancy (RC = Disagree)	
Agree	1.84**** (1.33–2.53)
Unmarried women or girls should have access to contraception (RC = Disagree)	
Agree	2.15**** (1.61–2.88)
Married women or girls should have access to contraception (RC = Disagree)	
Agree	1.55 (0.99–2.39)
Women know where to obtain contraception for prevention against pregnancy (RC = Disagree)	
Agree	2.35** (1.19–4.65)
Women who carry condoms are promiscuous (RC = Disagree)	
Agree	0.92 (0.70–1.20)
It is not good for girls to know about sexual matters because they will get spoilt (RC = Disagree)	
Agree	0.79 (0.62–1.02)
District of study (RC = Amudat)	
Kaberamaido	2.58** (1.23–5.39)
Kasese	2.62** (1.25–5.47)
Moroto	0.27*** (0.11–0.68)
Pader	4.35**** (2.15–8.79)
Tororo	9.44**** (4.59–19.37)
Constant	0.03**** (0.01–0.09)
Number of observations	2230
Likelihood Ratio Chi-squared (Probability)	713.23 (0.000)
Pseudo R-squared value	0.279

Note: **=*p* < 0.05; ***=*p* < 0.01; ****=*p* < 0.001. Missing cases are not shown. Figures may not add up to 100 % due to rounding errors

## Discussion

This study demonstrates a relatively high proportion of respondents having negative attitudes in relation to access to contraceptive services for adolescents. This is in line with other studies that have generated similar results [21, 22, 29]. Similarly, other studies have shown that prevalent of negative attitudes towards women/girls carrying condoms, sexuality education for girls, and unmarried women or girls’ access to contraception in low income settings [4, 22, 24].

Our study shows that it is not acceptable for adolescent girls in the community to access contraceptives This shows that this norm is widely shared in the communities in our study sites and this has implications for access to adolescent sexual and reproductive health services especially contraceptives [30, 37]. Further, given the significant differences in relation to this social norm—“girls are not allowed to access contraception in this community” by age, marital status, level of education and religion, programmes engaged in promoting SRHR particularly for adolescents must pay attention to differences in beliefs in the various age groups, among those who married and the unmarried as well as pay attention to the differences in level of education. This points to need for audience segmentation in designing social norm change and social behavioural change communication strategies and messages to address the heterogeneity of the population in the study sites.

Education was generally associated with supporting progressive or positive norms towards allowing adolescent girls to access contraception in the community. Other studies have also shown similar findings that formal education tends to influence attitudes and social norms positively in relation to access and use of contraceptives by adolescent girls in the communities [57–60].

Our study demonstrates that most respondents had a negative attitude towards girls’ access to adolescent sexual and reproductive health (ASRH) services especially contraception. These attitudes further reinforce non supportive norms in relation to ASRH services particularly those linked to adolescent girls being allowed to access contraception in the communities that have been established by other studies [16, 23, 41].

Being in formal employment was associated with progressive norms towards adolescent girls accessing contraception in the community. This suggests that having a source of livelihood or being economically empowered through employment increases agency to challenge prevailing negative norms that regulate behaviour in related to access of contraception for girls.

Religion, particularly being of Christian religion was being less likely to support adolescent girls in the community to have access to contraception [61]. Some scholars have argued that religious teaching in Christian faith especially the Catholic Church tends to discourage the use of modern contraceptives because they encourage promiscuity [62]. However, the effect of education, marital status and religion fall away when all factors are controlled for in the full model.

Generally, attitudes had a strong bearing on social norms related to access to adolescent sexual and reproductive health among the study sites. Respondents who had positive beliefs towards adolescents accessing contraception were more likely to agree with the social norm that adolescent girls are allowed to access contraception in the community. Similarly, respondents who had negative or non-progressive beliefs about adolescents’ access to contraception were less likely to agree that adolescent girls in the community are allowed to access contraception. This is similar to previous studies that link attitudes to social and gender norms that regulate sexual behaviour and adolescent use of contraception [63–66].

Geographical location or cultural context seemed to influence the likelihood that respondents would support a positive norm that adolescent girls are allowed to access contraception in the community. For example, the likelihood for adolescent girls to be allowed access to contraception in the community was higher in Kaberamaido, Kasese, Pader and Tororo but lower among respondents living in Moroto and Amudat all found in Karamoja sub-region – which is categorized as one of the least developed regions in Uganda [42, 43]. This implies that cultural context in the different districts plays a role in shaping social norms and attitudes related to access to contraception for adolescent girls [38, 39].

## Limitation

While every effort was made to ensure representativeness, respondents who stayed at home for the entire period the survey was conducted might not be well represented. Nonetheless, the data used in this paper provides an excellent snapshot of what is happening in the community.

## Conclusions

Overall, our study demonstrates that a higher proportion of respondents hold social norms that inhibit adolescent girls from accessing contraception in the community. This is reinforced by negative attitudes towards allowing girls to access and use adolescent sexual and reproductive health services especially contraceptives. Those who believe in effectiveness of traditional methods of contraception tended to support social norms that inhibit girls from accessing contraception in their communities. Cultural context and location had an influence on supporting social norms that do not allow adolescent girls to access contraception—respondents from districts in Karamoja sub region (Moroto and Amudat) were more likely to report social norms and attitudes that inhibit adolescent girls from accessing contraception. Our findings suggest the need to apply social norm change approaches in addressing social norms and attitudes that inhibit access and use of contraception by adolescents in areas where adolescents are prone to early/unwanted pregnancies and other negative reproductive health outcomes such as sexually transmitted infections, including HIV in Uganda and other low income countries in Africa. Our findings also underscore the need for context specific ASRH programs that take into account the differences in risk factors, attitudes and social norms that affect access and use of contraception by adolescents.

## Data Availability

The datasets used in this study are available from the corresponding author on request.

## Abbreviations

ASRH: Adolescent Sexual and Reproductive Health
HP: Harmful Practices
SRHRs: Sexual and Reproductive Health Rights
UBOS: Uganda Bureau of Statistics
UN: United Nations
UNDP: United Nations Development Programme
UNICEF: United Nations Children’s Fund
VAWG: Violence Against Women and Girls
